# The majority of skin lesions in pediatric primary care attention could be managed by Teledermatology

**DOI:** 10.1371/journal.pone.0225479

**Published:** 2019-12-02

**Authors:** Mara Giavina Bianchi, Andre Pires Santos, Eduardo Cordioli

**Affiliations:** Department of Telemedicine, Hospital Israelita Albert Einstein, São Paulo, Brazil; San Gallicano Dermatologic Institute, ITALY

## Abstract

**Background:**

Teledermatology is a tool that provides accurate diagnosis and has been gaining more emphasis over time. It can be used for triage in primary care attention to address skin conditions improving access and reducing time to treatment for surgical, severe or even lethal diseases.

**Objectives:**

Our main goal was to evaluate the proportion of pediatric patient’s lesions that could be managed using teledermatology in primary care attention. Secondly, we wanted to assess the ten most frequent skin conditions, the most common treatments and the referrals made by the teledermatologists to biopsy, in-presence dermatologist or kept at primary care attention.

**Methods:**

A cross-sectional retrospective study involving 6,879 individuals and 10,126 lesions was conducted by store-and-forward teledermatology during one year in the city of Sao Paulo, Brazil. If the photographs taken had enough quality, teledermatologist would diagnose, treat and orient each lesion (if possible), and choose one of three options for referral: direct to biopsy, in-presence dermatologist or kept at primary care attention.

**Results:**

Teledermatology managed 62% of the lesions to be kept at primary care attention, 37% were referred to dermatologists and 1% to biopsy, reducing the mean waiting time for an in-presence visit in 78%. In patients 0–2 years old, lesions related to eczema and benign congenital lesions predominated. From 3–12 years old, eczema was still a major cause of complaint, as well as warts and molluscum. From 13–19 years old, acne was the most significant problem, followed by atopic dermatitis, nevi and warts. The most frequent treatment was emollient.

**Conclusion:**

Teletriage addressed 63% of the lesions without the need for an in-presence visit, suggesting that teledermatology can manage common diseases and optimize dermatological appointments for the most serious, surgical or complex skin illnesses, reducing the mean waiting time for them.

## Introduction

Teledermatology (TD) is a healthcare tool that has been more and more used around the world, mostly because Dermatology has an emphasis on visual diagnosis. Real-time (RT) or store-and-forward (SF) are the most common types of delivering images. In SF-TD, the data and images from a patient are collected and sent to a dermatologist analysis at a later time. In RT-TD, patients and physicians communicate data and images from separate locations in a live format [1]. Many studies have shown to improve access to specialized care using store-and-forward TD, which provides accurate diagnosis and reduces time to treatment, with high patient satisfaction [2].

Typical skin conditions such as mild atopic dermatitis, acne, fungal infections and others are common diseases that would be manageable within the primary care attention service. However, it is not what often happens because the professionals are not well trained to diagnose or triage skin diseases. The lack of primary care professional medical specialization can lead to refer individuals to dermatologists unnecessarily. If there is a shortage of dermatologists, this creates a situation where appointments are filled by patients who do not need special care, which can limit the availability of visits for those who do [3].

The city of Sao Paulo has nearly 12 million inhabitants [4], and 58% of them depend exclusively on the public health care system [5]. The instances of public health in Brazil are municipal, state and federal. Public municipal health care is in charge of most primary care attention. There is a high demand for a public dermatological consultation in the city of Sao Paulo. By July 2017, 57,832 individuals were waiting for it, which could take up to one year to occur. For this reason, the municipal health department has decided to implement a project of Teledermatology, in conjunction with Hospital Israelita Albert Einstein, a large private hospital in the city. The project rationale was: if the general doctor could manage some not so complex diseases with the teledermatologist support, more dermatological appointments would be available, shortening the waiting time for those patients who do need in-presence dermatological care.

It also is critical for public health to know the prevalence and management of the most common diseases presented at primary care attention, so policies can address or even prevent them. Our research had the primary objective to determine the proportion of pediatric patient’s lesions that could be managed at primary care attention through teledermatology, avoiding in-presence visits with dermatologists and, secondly, we assessed the frequency, treatment and referrals of the most common skin lesions in children and adolescents assisted in this project. Both goals were achieved in this study.

## Patients and methods

This study was approved by Hospital Israelita Albert Einstein and Municipal Ethics Committees (CAAE:97126618.6.3001.0086) and it is in accordance with the ethical standards on human experimentation and with the Declaration of Helsinki. Data was fully anonymized before accessed and the IRB waived the requirement for the informed consent. It was a cross-sectional retrospective cohort conducted in the city of Sao Paulo, where 14,925 individuals up to 19 years old had been waiting for an appointment with a dermatologist in July 2017. The municipal health government, in conjunction with Hospital Israelita Albert Einstein, developed a platform and an app for mobile device to be used by health technicians to take photographs with the its digital camera and upload them along with a short clinical history and patient data. All data were collected and uploaded into a platform accessed only by dermatologists recruited for this project using a secured online process. Patients in the waiting list were phoned by the public health care service and scheduled to go to an appointment in one of three public city hospitals enabled to carry out the project. From July 2017-July 2018, thirteen dermatologists worked in the triage of the patients and they had first to decide if the photographs taken of the lesions were satisfactory for diagnostic purposes. If not, they would choose the box "bad photo" in the platform and referred the patient to a face-to-face appointment with a dermatologist. If the photo had a good quality, they should formulate the most probable diagnostic hypothesis and choose among three referral options for each lesion assessed: 1) directly to biopsy (and after that patient would return to a dermatologist appointment with the result), 2) to a dermatologist visit or 3) back to the pediatrician in primary care attention with the most probable diagnosis and treatment and/or orientation how to proceed with the investigation or management of the lesion. If the same patient had more than one lesion with different referrals, a biopsy referral would prevail over dermatologist referral, that would prevail over back to pediatrician referral. All patients who attended the project were included in this study. For better analyze this population, we divided it into 0–2 years old, 3–12 years old and 13–19 years old. Only dermatologists certified by the Brazilian Board of Dermatology participated in the project in order to decrease the chance of diagnostic error by teledermatology. In our Municipal Basic Health Unit, the direct exam to search for fungal infection is not done. For this reason, suspicious cases of superficial fungal infections receive anti-fungal treatment as therapeutic test and are reevaluated afterwards. If deep fungal infections were suspected, then t the teledermatologist would refer the patient to Biopsy/Dermatologist and not treat him/her before the confirmation of the pathogen. All statistical calculations were done using two-tail Chi-square with Yates’ correction test by Graph Prism 6.0 software.

## Results

### 1) Patients and lesions

From 14,925 patients between 0–19 years old waiting for dermatologist consultation in the city of Sao Paulo, 6,879 individuals participated in this project (46%) and 6,588 referrals were made. Table 1 shows patients demographic data. There were more female than male individuals waiting for a consultation with a dermatologist in all ages studied. As the age increased, the presence rate increased from 37% at 0–2 years old to 47% at other ages. Patients referred to a dermatologist because of the poor quality of photographs totalized 1.4% and a small part of the patient data went missing due to technical problems (0.4%).

**Table 1:** Number of lesions photographed and individuals from 0 to 19 years old waiting for a dermatologist consultation and those who participated in the Teledermatology project, according to age and sex, from July 2017-July 2018, in the city of São Paulo.

	0–2 years old(n)		3–12 years old(n)		13–19 years old(n)		Total(n)
Patients	Female	Male	Female	Male	Female	Male	
Waiting	643	722	3674	3062	4169	2645	14925
Participating	239	271	1723	1419	1952	1275	6879
% of participation	37	38	47	46	47	48	46
Lesions photographed	327	370	2503	2103	3346	2009	10658
Lesions photographed per person	1.4	1.4	1.5	1.5	1.7	1.6	1.6
Poor quality photograph	3	2	25	22	27	14	93
Missing data	20	16	73	74	58	50	291

The total number of lesions photographed was 10,658 and 10,126 diagnostics were made. The mean number of lesions photographed per person was 1.6 (Table 1).

Bleeding was present in only 16% of the lesions, but pruritus was relatively common: 45–55% depending on age, being more frequent in lesions from individuals 3–12 years old. Lesions referred to a dermatologist because of the poor quality of photographs totalized 0.9% and missing data due to technical problems was 0.5%.

### 2) Referrals and most frequent diseases

**Fig 1:**
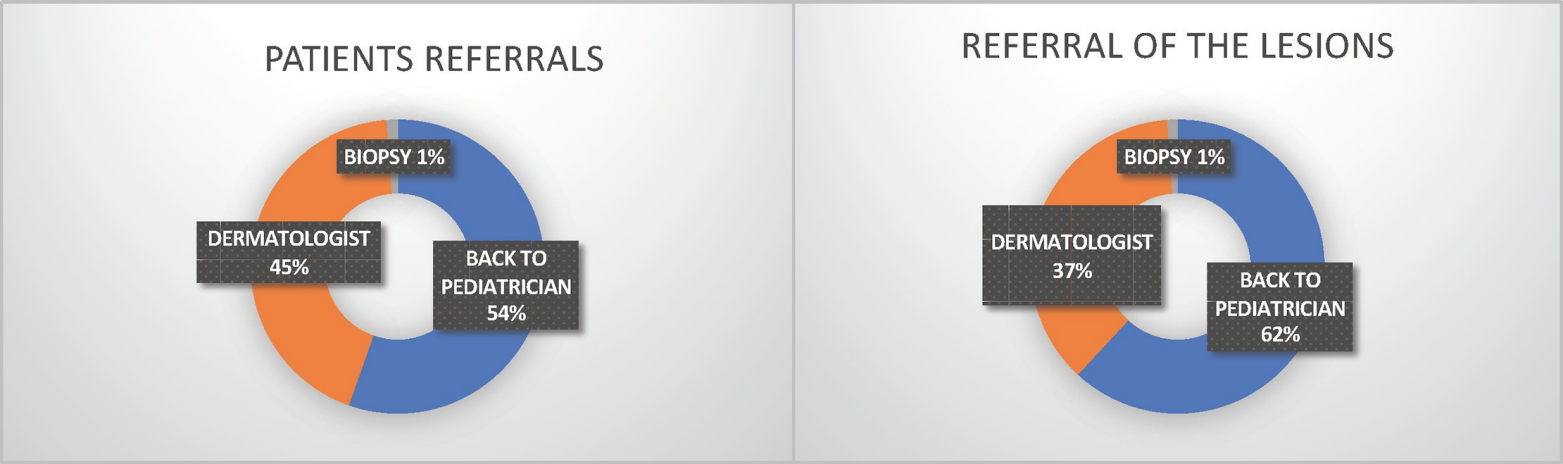
Distribution of the lesions and the patients 0–19 years old who participated in the Teledermatology Project referred to biopsy, dermatologist and back to pediatrician according to age and sex, from July 2017-July 2018, in the city of São Paulo.

Considering patients, referrals back to their primary care pediatrician occurred in 54%, to the in-presence dermatologist in 45% and directly to biopsy in 1%. If lesions are analyzed, referrals back to their primary care pediatrician were 62%, to the in-presence dermatologist 37% and directly to biopsy 1%. (Fig 1). The use of Teledermatology reduced the mean waiting time for in-presence dermatologist visit from 6.7 to 1.5 months during the time of the project (78% reduction).

**Fig 2:**
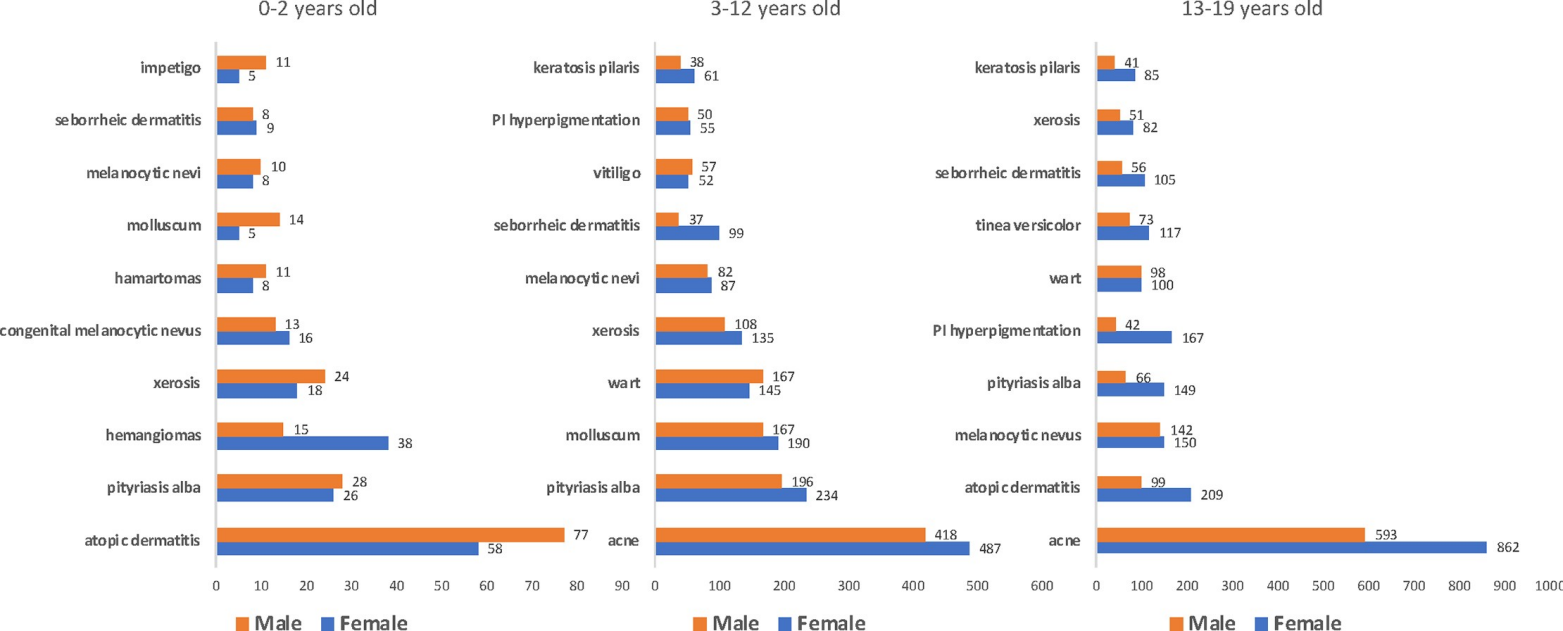
Most frequent diseases in patients 0–19 years old who participated in the Teledermatology Project according to age and sex, from July 2017-July 2018, in the city of São Paulo.

The most common causes of consultation for male and female individuals up to 19 years old is shown in Fig 2. Diseases frequency varied along the ages. Among patients 0–2 years old, the most common ICD-10 code for lesions was atopic dermatitis (AD) for both sexes. Boys were more affected than girls, but with no statistically difference. Other complaints associated frequently with AD, pityriasis alba and xerosis were also very frequent, summing 37%. Another frequent cause of consultation was benign tumors, such as hemangiomas, congenital nevus and hamartomas (verrucous nevus, sebaceous nevus, café-au-lait macule and spilus nevi) which together with acquired melanocytic nevus, accounted for nearly 20% of the cases. Hemangiomas were statistically more frequent in girls than in boys (p = 0.0002). Infectious causes as molluscum contagiosum and impetigo totalized around 5–6% and seborrheic dermatitis complete the top ten ICD-10 codes with 3% of the lesions.

From 3–12 years old, AD maintained the first position on the list, as the conditions related: pityriasis alba, xerosis and keratosis pilaris, summing up to 40%. Congenital malformations no longer represented a complaint in that age. Only acquired melanocytic nevus was among the top complaints, with 3%. Infectious viral diseases, such as molluscum and wart, showed a great increased (15%) and impetigo continued to be the 10^th^ most common cause of visits, around 2%. Seborrheic dermatitis still accounted for 3% and pigmentation disorders such as vitiligo and post-inflammatory hyperpigmentation start to appear as frequent causes of consultation, around 5%.

For individuals 13 to 19 years old, acne is the greatest complaint, with almost 30%. AD decreases to second place, with 6%. Pityriasis alba and xerosis also decreased by around half, but, together with keratosis pilaris remain at the top ten causes. Nevi and seborrheic dermatitis maintained almost the same percentage and molluscum disappeared from the ten most frequent complaints. Warts, in the other hand, kept its place, although less frequent. Post-inflammatory hyperpigmentation is on a rise in that period, and fungal infection, such as Tinea Versicolor, starts to be a frequent motive of consultation (3–4%).

**Fig 3:**
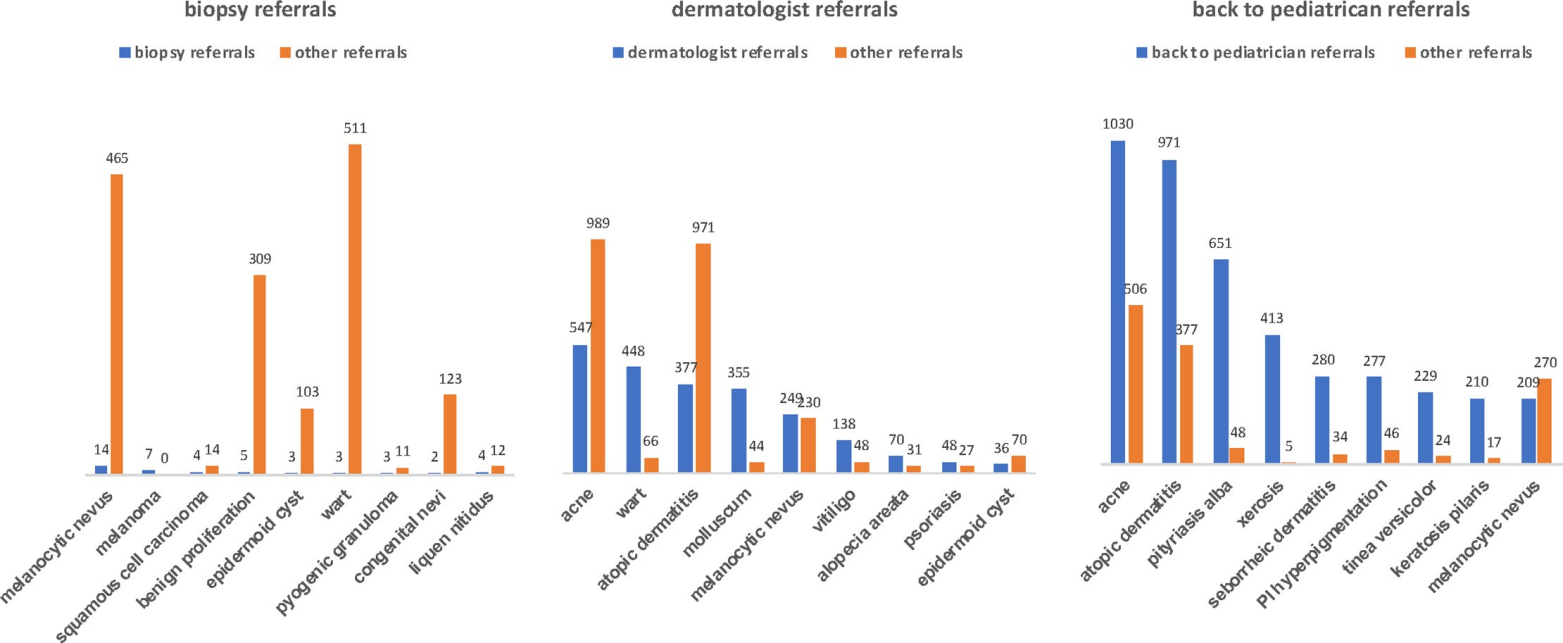
Most frequent lesions sent to biopsy, dermatologists and back to pediatrician in patients 0–19 years old who participated in the Teledermatology Project according to age and sex, from July 2017-July 2018, in the city of São Paulo.

The most frequent lesions referred to biopsy, dermatologist and back to the primary care pediatrician is shown in Fig 3. Biopsy was chosen in only 1% of all cases. Benign tumors were the most frequent cause: melanocytic nevus, benign neoplasm such as soft fibroma, acrochordon, pyogenic granuloma, epidermoid cyst and congenital melanocytic nevi totalized 27 cases. However, it was indicated in only a small percentage (around 3% or less). The only situation where a suspect of a benign lesion had more referrals to biopsy was a pyogenic granuloma with 21%. For malignant tumors, such as melanoma, the second most frequent lesion biopsied, it was indicated every time (100%). Wart was also rarely biopsied (0.6%). Lichen nitidus was the third most common disease to be biopsied, with 4 cases in 25% of the times.

Referrals for dermatologist occurred in 37% of the lesions. Acne and AD were frequent causes, but only in 35% and 28%, respectively. Infectious diseases that often need in-presence treatment, such as warts and molluscum, were sent to dermatologists in almost 90% of the times. Benign lesions such as melanocytic nevus and epidermoid cyst were sent to the specialist in 52% and 34% of the cases. Vitiligo, alopecia areata and psoriasis were common diseases referred to a dermatologist (74%, 69% and 64% respectively).

Teledermatologists sent the patients back to their pediatricians in 62% of the all cases. For lesions of acne and AD, around 70% of the times, and >85% for Pityriasis alba, xerosis, seborrheic dermatitis, hyperpigmentation post-inflammatory hyperpigmentation, Tinea Versicolor and impetigo. The most significant exception was melanocytic nevus, with only 44% of the referrals back to their doctors.

### 3) Treatment

**Fig 4:**
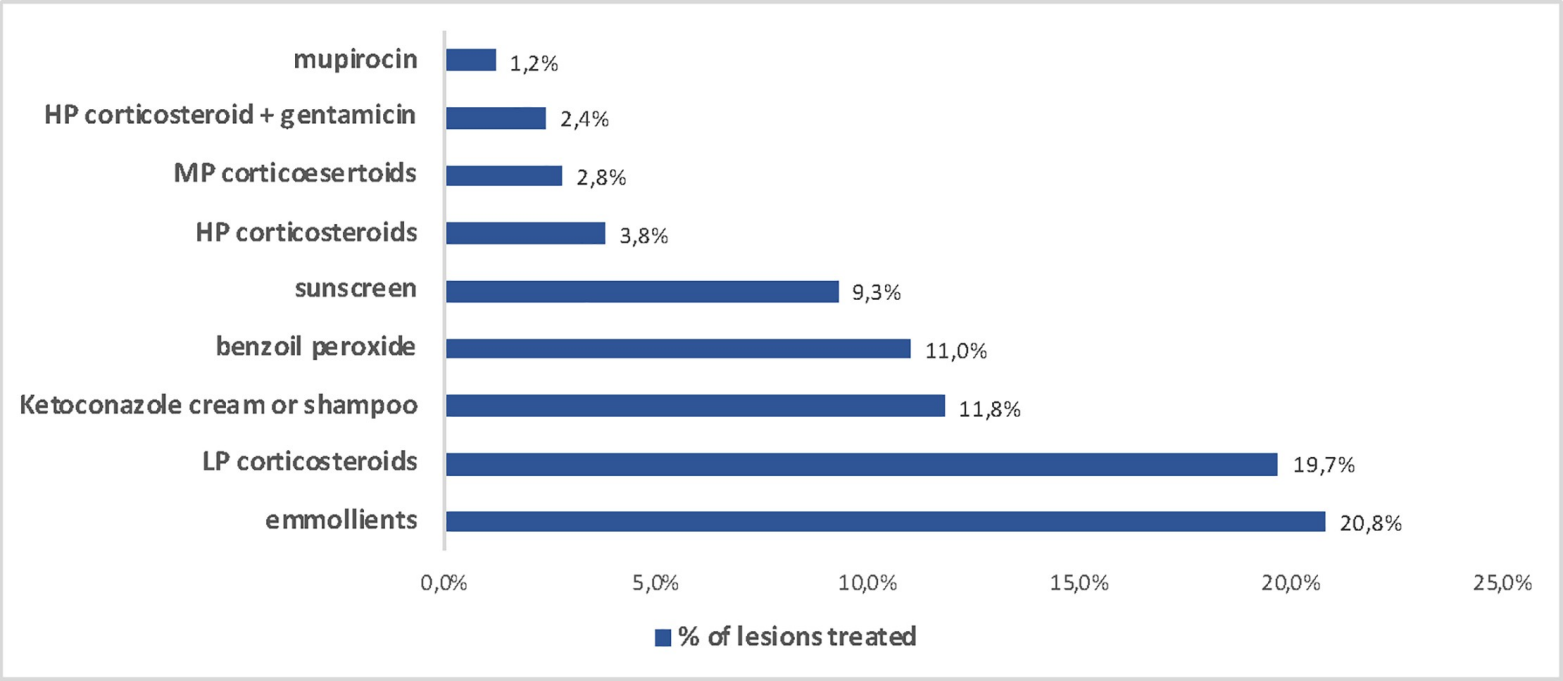
List of most frequent prescriptions made by teledermatologists for patients 0–19 years old who participated in the Teledermatology Project from July 2017-July 2018, in the city of São Paulo.

We also searched for the most frequent treatments prescribed by teledermatolgists (Fig 4). All products in the top ten list were topical. Emollients were the most frequent prescription in 32% of all cases. Topical corticosteroids combined summed 29%, topical antifungal 12%, topical anti-acne 11%, sunscreen 9% and topical antibiotics 4%.

## Discussion

In our view, the presence of 47% of the individuals called for this project was reasonable. TD is still very new in our country, both for patients and physicians, and a pioneer project like this, involving a significant number of people was a challenge. Our TD work had the benefits of a large cohort and virtually any type of skin disease covered. Most of the TD articles focus on one disease (melanoma) or a class of diseases (malignant tumors) [6–8]. In this way, we did find our study very interesting and innovative.

Most of the lesions did not bleed, but pruritus was frequent, in nearly 50% of the cases. The vast majority of the lesions photographed in our project did not require an in-presence visit with the dermatologist (63%). This proves that most skin conditions in our primary care setting is of low complexity and, therefore, can be properly addressed in primary care. This finding reinforces the feasibility and the importance of Teledermatology in this context, helping the pediatricians to manage such conditions and optimizing medical hours and costs, especially in the public system. The mean waiting time for an in-presence visit with the dermatologist was reduced 78% during the project (from 6.7 to 1.5 months). Our teledermatology project proved to be a well-defined, structured, scalable process with standardized collection and fairness of care, which might promote the democratization of access to dermatology for underprivileged patients. Another study with 850 consultations showed that in 50% of the patients no referral to an in-person dermatologist was needed [3]. Avoiding face-to-face consultations with dermatologists in cases of frequent and manageable diseases by teledermatology can optimize access to more severe, surgical or complex illnesses, which do require the presence of a dermatologist. One important limitation of our work is the chance of error and bias in teledermatology diagnosis, although many articles have attested a high agreement rate between teledermatology and in-presence dermatology [6–9].

Most of the referrals for biopsy were benign lesions, but only in a small fraction of them (1–25% depending on the suspected diagnosis), but 100% all suspects of malignant lesions went for biopsy. Prevalent diseases, such as acne and atopic dermatitis, do not need biopsy confirmation to be managed. In typical lesions, this procedure might also has been avoided because of the difficulty inherent for the age: younger patients may have to undergo sedation or general anesthesia, which carry risks, and children, most of the times, do not collaborate in the procedure, turning it into a stressful situation for the them and their relatives.

Referrals to dermatologists were made mostly for treatment of infectious diseases such as warts and molluscum and for conditions that require follow up, such as vitiligo, psoriasis and alopecia areata. Probably, most of the cases of acne and atopic dermatitis referred to dermatologists were moderate to severe, since the vast majority of the cases of those two diseases was sent back to the pediatrician after being addressed. Benign neoplasms such as nevus and cyst were also referred to the dermatologist, mostly because it is advisable to look at all nevi with dermoscopy (that was not available in our project). If we had included it in the project, the rates of referral would be much smaller [10]. For cysts, dermatologists are trained to palpate such lesions, so teledermatology might feel insufficient in some cases.

Teletriage diagnosis for children 0–12 years old was mostly associated with atopic dermatitis and commonly associated skin disorders, such as pityriasis alba and xerosis (32%). Other causes were benign lesions such as melanocytic nevus and congenital malformations (hemangiomas, verrucous nevus, sebaceous nevus, café-au-lait macules) and infectious diseases such as warts and molluscum. Once patients enter adolescence, acne was the primary cause of complaint (28%), along with AD, melanocytic nevi, post-inflammatory hyperpigmentation and warts. When we compare our data to other prevalence studies, atopic dermatitis was around 20% in children up to 12 years old and 6% of adolescents as other studies have shown [11,12]. Pityriasis alba is also very common, in our study ranging from 4–10% of all lesions photographed, compatible with another study that showed 4–7% of the school students in Turkey [13]. This same study found that nevi are one of the most frequent dermatosis, around 60–70% [13]. Melanocytic nevi were the primary cause for the teleconsultation in 4–7% of the individuals up to 19 years old in our project. Another teletriage study showed nevi as 14% of their cause for consultation [14]. Hemangiomas are a common tumor of the infancy, and its prevalence is said to be likely around 5%, according to one study [15]. Our project has shown that hemangiomas were 13% of the complaints in girls and 5% in boys between 0–2 years, which was statistically significant (p = 0.002), in a proportion of almost 3:1, which in accordance to the literature [16].

Cross-sectional study completed in schools has shown warts to have a prevalence between 2 and 20% in children [17]. We have encountered around 6–8% in children 3–12 years old and 3–5% from 13–19 years old. Molluscum contagiosum meta-analysis suggests a point prevalence in children aged 0–16 years of 5–11% [18]. In our study, it was 3% and 8% of the cases in children from 0–2 and 3–12 years old, respectively. Although the true prevalence of post-inflammatory hyperpigmentation is not known, it is widespread among Fitzpatrick types IV-VI, but not so much among Caucasian patients [19]. Our data showed 2–5% of the complaints in children from 3–19 years old. Several extensive studies have reported a prevalence of adolescent acne ranging from 81 to 95% in young men and 79 to 82% in young women [20]. Our project found acne to be around 30% of the causes to have the teleconsultations. Around 6% of the school students presented xerosis in one study [13] and 7%, 5% and 3% of the children between 0–2, 3–12 and 13–19 years old in our research.

Regarding treatment, a list of which drugs were available to the population studied is found in S1 Table. All physicians involved were encouraged to use as much as possible only medications from such list, once they are free for patients and, most of them are unable to buy them otherwise. The results show the importance of emollients in skin conditions, prescribed in 1/3 of the times. Along with other studies that support this, we could suggest that pediatricians prescribe them routinely for most of the patients, as treatment and prevention of common skin diseases, such as AD, and xerosis [21,22]. Low potency corticosteroids were the second most prescribed medication, but moderate and high potency corticosteroids also played an important rule being in the top ten list. Ketoconazole cream or shampoo for superficial fungi infections and seborrheic dermatitis was very frequently recommended, as benzoyl peroxide for acne. Sunscreen is essential in Brazil, especially for post inflammatory hyperpigmentation in our dark skin population. Drugs containing antibiotics were also in the top ten list, mostly for secondary infected lesions or impetigo.

There are limitations for Teletriage in Dermatology. The fact that you can receive multiples photographs of parts of the body and head, and not to examine the body as a whole, makes the diagnosis more challenging. Also, some important impressions that would help to corroborate the diagnosis, such as feeling the texture of the skin or proceeding easy tests (p.e. vitrocompression) cannot be done. However, we have shown that the impossibility to palpate the lesion is not a main issue to the teledermatologists, who became much more confident in Teledermatology after working with TD [10].

The patients included in this project were referred to dermatologists by their pediatricians and if they were not referred to a face-to-face dermatologist by the Teletriage project, the patients went back to their pediatricians, who received information, recommendations and management for that disorder. Therefore, there was not a moment when the patient was not supervised by a physician and we believe that this fact gave us more confidence that potentially severe, life-threatening or rare diseases were not being overlooked. Also, if any other concern was raised, there was always the possibility of referring the patient to the dermatologist again. Sometimes, besides the written concern that moved the pediatrician to refer the patient, while taking photographs, the parents could spontaneously ask the health technician to include some other lesions(s), which occurred sometimes, and the teledermatologists would address those complaints too.

## Conclusions

Teledermatology managed 63% of the lesions without the need for an in-presence appointment with the dermatologist, keeping 62% of them with their own primary care physician and referring 1% directly to biopsy. Only 37% of the lesions were referred to dermatologists, optimizing consultations for the most serious, surgical or complex skin illnesses, which do require the presence of a dermatologist. Its use reduced the mean waiting time for these later patients from 6.7 months to 1.5 during the time of the project (78% reduction). Teledermatology does not aim to replace face-to-face visit with the dermatologist, but it has been proving to be an efficient tool for triage and management of less complex dermatosis together with the primary care pediatrician.

The frequency of skin diseases differed according to age. From 0–2 years old, lesions related to eczema predominated along with benign congenital lesions. From 3–12 years old, eczema was still a major cause of complaint, as well as warts and molluscum. From 13–19 years old, acne was the most significant problem, followed by AD, nevi and warts. The most prescribed product was emollient in 1/3 of the cases.

## Supporting information

S1 Table List of most common disease included into the disease groups. (DOCX)
